# Alteration of hemoglobin ß gene expression in mucosal tissues of Japanese flounder, *Paralichthys olivaceus*, in response to heat stress, *Edwardsiella piscicida* infection, and immunostimulants administration

**DOI:** 10.1016/j.fsirep.2021.100049

**Published:** 2022-01-08

**Authors:** Misato Mori, Yasuhiro Shibasaki, Aki Namba, Takeshi Yabu, Noriko Wada, Hajime Shiba, Hirosi Anzai, Nobuhiro Mano

**Affiliations:** College of Bioresource Sciences, Nihon University, Graduate School of Bioresource Sciences: Nihon Daigaku Seibutsu Shigen Kagakubu Seibutsu Shigen Kagaku, Kameino 1866, Fujisawa, Kanagawa 252-0880, Japan

**Keywords:** Hemoglobin beta, Edwardsiella piscicida, Heat stress, Immunostimulant, Health status, (AsA), Ascorbic acid, (Hbß), Hemoglobin beta, (LF), Lactoferrin

## Abstract

Hemoglobin beta (Hbß) is a heme-binding protein capable of oxygen delivery. The oligopeptides derived from Hbβ in fish mucus are active against a variety of gram-negative bacteria and protozoa. To gain information on the physiological and immunological roles of Hbβ in the mucosal tissues of fish, we analyzed changes in Hbß gene expression levels in the epidermis, gills, and intestine of Japanese flounder, *Paralichthys olivaceus*, in response to heat stress, *Edwardsiella piscicida* infection, and trial feeding of immunostimulants, high-concentration ascorbic acid (AsA) or lactoferrin (LF). The results of quantitative real-time PCR showed that expression of the Hbß gene in the gills decreased markedly when exposed to heat stress, whereas that in the epidermis exhibited an increase 3h after infection with *E. piscicida*. Seven days after starting to feed either immunostimulant, epidermal Hbß gene expression in all AsA or LF dose groups was significantly higher than in the control group. The results of *in situ* hybridization showed that the abundance and intensity of the stained cells in the epidermis and in the gills were consistent with the expression levels of Hbß gene obtained from the infection and immunosuppressant experiments and the heat stress experiment, respectively. Our results suggest that mucosal Hbβ gene expression is closely related to physiological and immunological status and could be a useful indicator for monitoring condition of fish health.

## Highlights

• Hbβ gene expression in the gills is decreased upon heat stress.
• Epidermal Hbß gene expression was increased upon AsA and LF feeding or infection.
• Mucosal Hbβ expression may be a useful indicator for monitoring fish health status.

## 1 Introduction

In fish and mammals, hemoglobin, a heme-binding protein, is a tetrameric protein consisting of two α and two ß chains [1]. Its primary function is to deliver oxygen to tissues [2]. It is also known as a source of oligopeptides with several biological functions, such as growth-hormone-releasing activity [3], opioid-like activity [4], and antimicrobial activity [5]. The antimicrobial activity of the C- and N-terminal peptides derived from the hemoglobin and ß chain (Hbß) against a variety of gram-negative bacterial species [[6],[7]] and protozoa (such as *Ichthyophthirius multifiliis* [6]) suggests that these antimicrobial peptides are important defense factors in fish.

In a previous study, we fed Japanese flounder, *Paralichthys olivaceus*, a diet supplemented with high-concentration ascorbic acid (AsA) as an immunostimulant. We found that Hbß was induced in the skin mucus and that Hbß gene expression was markedly increased in the epidermal cells [8]. Immunostimulants are capable of enhancing the non-specific immune response [9]. In fish, immunostimulant feeding increases resistance to several types of stress, e.g., handling [10] and high temperature [11] and helps to maintain good health [[12],[13]].

Although there is little information on the correlation between health status and expression or secretion of antimicrobial molecules so far, we hypothesized that Hbß levels could be used as an indicator for monitoring disease and the stress status of fish. In the present study, we analyzed changes in expression of the Hbß gene in three mucosal tissues (ocular-side epidermis (epidermis), gills, and intestine) of Japanese flounder under three different experimental conditions, i.e. heat stress, *Edwardsiella piscicida* infection, and immunostimulant administration.

## 2 Material and methods

### 2.1 Experimental materials

Japanese flounder *Paralichthys olivaceus* (5.99 ± 1.45 g) were purchased from Marinetech Co., Ltd., Aichi, Japan. They were acclimated in a stock aquarium at 20°C for 2 weeks and fed a commercial diet (hirame EP F-3, Nissin Marubeni Feed Co., Ltd.) at 3% body weight daily. *Edwardsiella piscicida* HH-1 was cultured according to the method described by Miwa and Mano [14], with a slight modification. Briefly, *E. piscicida* was inoculated onto heart infusion (HI) agar containing 1% NaCl and incubated at 20 °C for 48 h. Subsequently, the bacteria were harvested, suspended in 200 mL of HI broth containing 1% NaCl, and cultured with shaking at 20 °C for 18 h. The number of bacteria (CFU/mL) was quantified by serial dilution of the inoculum on HI agar. L-ascorbic acid (AsA: guaranteed reagent) and bovine lactoferrin (LF: 90% purity) as immunostimulants were purchased from FUJIFILM Wako Pure Chemical Corporation, Osaka, Japan and from Morinaga Milk Industry Co. Ltd., Tokyo, Japan, respectively. Experimental diets (0, 500, 1000, 2000, 5000, or 10,000 mg AsA and 0, 100, or 1000 LF equivalent/kg diet, designated AsA0, AsA500, AsA1000, AsA2000, AsA5000, AsA10,000 and LF0, LF100, LF1000, respectively) were prepared by mixing the commercial diet and distilled water containing AsA or LF at the relevant concentration and stored at –20 °C. The AsA contents of the experimental diets was analyzed by high-performance liquid chromatography following a previous study [[15],[16]] are shown in Table 1.

**Table 1:** Experimental designs of this study.

Experiment		Group	No. of fish /aquarium	Body weight (g: means ±S.D.)	Water temperature (°C)	AsA content in diet (mg/kg diet)	AsA concentration in liver (mg/100g tissue)	Challenge dose (CFU/ml)	Analyzed gene name
Heat stress		Control	30	6.07±1.14	20	-	-	-	*hemoglobin beta*
		Heat			30				*heat shock protein 70*
*Edwardsiella piscicida* infection		Control	20	5.98±1.28	20	-	-	0	*hemoglobin beta*
		Infection						2.7×10⁵	
Immuostimulant	Ascorbic acid	AsA0	10	6.26±1.52	20	81	5.3±1.3 a	-	*hemoglobin beta*
		AsA500				621	9.0±0.6 b		
		AsA1000				1570	12.3±2.2 c		
		AsA2000				2746	19.2±4.2 d		
		AsA5000				5460	25.2±3.9 e		
		AsA10,000				11,117	30.0±6.6 e		
	Lactoferrin	LF0	10	5.63±1.87	20	-	-	-	*hemoglobin beta*
		LF100							
		LF1000							

Not measured. Values of AsA concentration in liver and body weight are means ± S.D. of each group. Data were statistically analyzed using the Tukey-Kramer multiple comparison tests. The letters indicate significant differences (*p* < 0.05) among groups.

### 2.2 Experimental designs

The three experiments, heat stress, bacterial infection, and immunostimulant administration, were performed at different times using different fish. The experimental designs are summarized in Table 1. In each experiment, fish were randomly selected from the stock aquarium. We used quantitative real-time PCR (qRT-PCR) and *in situ* hybridization (ISH) to quantify and localize Hbß expression, respectively. Details of the conditions and sampling procedures used for each experiment are given below.

#### 2.3.1 Heat stress

To perform the heat stress experiment, 60 fish were randomly allocated to two experimental tanks (200 L). In one group, the water temperature was raised by an aquarium heater (Nisso Protect heater R-500W, Marukan Co., Ltd., Osaka, Japan) from 20 °C to 30 °C (1 °C/h) and then kept at 30 °C for 72 h (heat group). The other group was kept at 20 °C throughout the experimental period (control group). At 0 (pre-stress), 3, 12, 24, 48, and 72 h of heat stress, five fish were sampled from each group and anesthetized with ethyl 3-aminobenzoate methanesulfonate salt (MS-222: 0.2 g/L, Sigma-Aldrich). Mucosal tissues (ocular- side epidermis, gills, and intestine) were removed and stored at –80 °C in RNAlater (Invitrogen, Thermo Fisher Scientific) for qRT-PCR or in OCT compound (Sakura Finetek Japan, Co., Ltd., Tokyo, Japan) for ISH.

#### 2.3.2 Edwardsiella piscicida infection

To analyze the effects of bacterial infection, 20 fish were immersed in an *E. piscicida* suspension (2.7 × 10^5^ CFU/mL) for 30 min (infection group). Another 20 fish were similarly immersed in heart infusion broth as the control group. After immersion, the fish were moved to experimental tanks (182 L) at 20 °C and maintained for 24 h. At 0 (pre-infection), 3, 12, and 24 h after infection, mucosal tissues were sampled from five fish in each group for qRT-PCR, and the skin tissues were sampled for ISH and treated as described above.

#### 2.3.3 Immunostimulant administration

To investigate the effects of different concentrations of each immunostimulant, a total of 90 fish were randomly assigned to nine experimental tanks (170 L) and an AsA or LF feeding trial was conducted at 20°C. The experimental diets were fed to the fish at a rate of 3% body weight daily, and the epidermis and liver were collected from five fish in each of the AsA groups on days 3 and 7 of the feeding trial and in each of the LF groups on days 7 and 14 of the feeding trial following previous reports [[8],[17],[18]]. All collected tissues were treated and frozen as described above. The liver AsA content was determined as described previously [16].

### 2.4 qRT-PCR

Total RNA extraction, DNase treatment, cDNA synthesis, and qRT-PCR analysis were performed according to our previous report [8]. Briefly, total RNA was extracted from the samples (20–30 mg of tissue) by using an Isogen II RNA extraction kit (Nippon Gene Co., Ltd., Tokyo, Japan). After DNase treatment by using a Heat Stop kit (Nippon Gene Co., Ltd., Tokyo, Japan), the concentration of total RNA was adjusted to 0.1 µg/µL as a template, and cDNA was synthesized by using a High-Capacity cDNA Reverse Transcription Kit (ThermoFisher Scientific, USA) in the presence of Qiagen RNase Inhibitor (Qiagen). All primers used in this assay are listed in Table 2 and supplementary Table. The ß-actin gene was employed as an internal control, and the relative expression levels of the target gene were calculated by using the 2^−ΔΔCt^ method where the mean value was obtained when normalized against the expression of the reference β-actin gene. All reactions were run in triplicate.

**Table 2:** Primers used for quantitative real-time PCR (qRT-PCR) and in situ hybridization (ISH).

Assay	Target	Gene abbreviation		Nucleotide sequence (5′-3′)	Reference
qRT-PCR	β-actin	β-actin	Forward Reverse	TTCCTCGGTATGGAGTCCTG AGCACAGTGTTGGCGTACAG	[8]
	Hemoglobin beta	Hbβ	Forward Reverse	TGCTCTGGGCAAACAGTACC TCGGGAAAGGTTTCTCTGCG	[8]
	Heat shock protein 70	HSP70	Forward Reverse	TTCAATGATTCTCAGAGGCAAGC TTATCTAAGCCGTAGGCAATCGC	[39]
ISH	Hemoglobin beta	*-*	Forward Reverse	ATGGTCCAGTGGTCAGCTTG AGCCTCAGTGGTACTGTTTGC	[8]

### 2.5 ISH

cDNA corresponding to the protein-coding region of Hbß (450 bp) was amplified by using a primer set (Table 2), and probes were then synthesized using a DIG [digoxigenin] RNA labeling kit (Roche, Switzerland).

ISH was performed according to a previous report [8]. Briefly, cryosections (5 or 10 μm) of skin (ocular side) and gill tissues were fixed in paraformaldehyde and then permeabilized by using Triton X-100 and acetylation buffer. After pre-hybridization using hybridization solution, hybridization was performed overnight at 50°C with hybridization solution containing 1.1 ug/mL of DIG-labeled antisense or sense probe. The signals were visualized by using anti-Digoxigenin-POD, Fab fragments (Roche) and a HistoGreen Substrate kit for peroxidase (Cosmo Bio. Co. Ltd., Tokyo, Japan). The slides were mounted with Entellan new (Merck) and observed under a light microscope.

### 2.6 Statistical analyses

Data are shown as means ± standard deviation. Results were analyzed by Tukey-Kramer multiple comparison tests, the Mann-Whitney U-test or Steel-Dwass test. Statistical significance was determined with *P* < 0.05.

## 3 Results

### 3.1 Heat stress

**Fig. 1:**
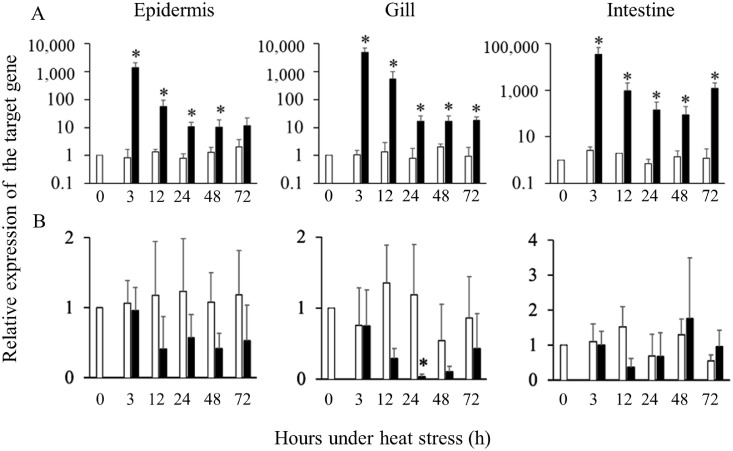
Changes in HSP70 (A) and Hbβ (B) gene expression levels in the ocular side of the epidermis, gill, and intestine of Japanese flounder under heat stress (30°C; closed bars) or control conditions (20°C, open bars). The gene expression levels in each tissue were determined by quantitative real-time PCR and were normalized to that of the housekeeping gene β-actin. Expression levels relative to the pre-stress control group are shown. Values are means ± S.D. (*n* = 5). * *p* < 0.05 versus corresponding control group at the same time point (Mann-Whitney *U* test).

**Fig. 2:**
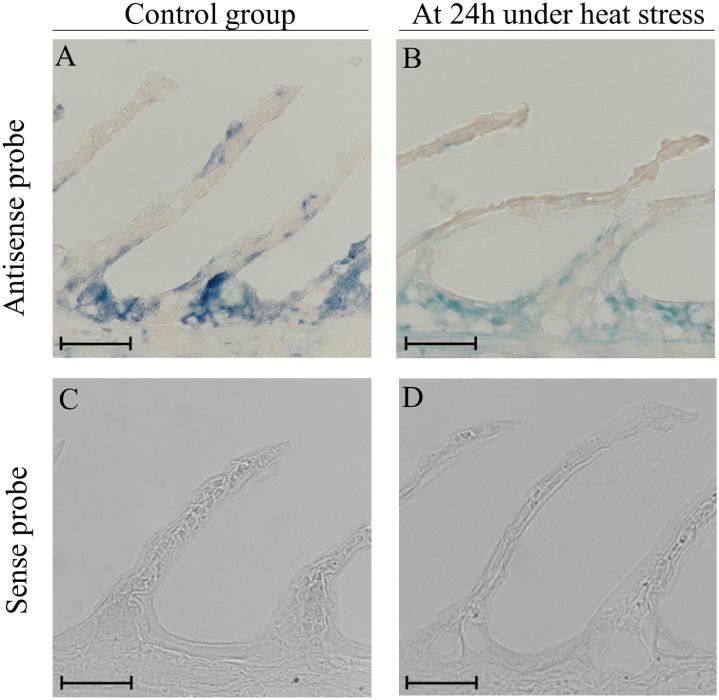
Expression of Hbβ mRNA (blue staining) in gill tissue of Japanese flounder under heat stress. The sections were prepared from gill in pre-stress control (A and C) and at 24 h under heat stress (B and D). *In situ* hybridization was performed by using the antisense probe (A and B) and the sense probe (C and D). Scale bars, 10um.

To evaluate the effects of heat stress, we first performed qRT-PCR analysis of heat shock protein 70 (HSP70). Throughout the period of experimental heat stress, HSP70 gene expression levels were significantly greater than in the corresponding control group in all mucosal tissues, except at 72 h in the epidermis (Fig. 1A). In contrast, Hbß gene expression levels in the epidermis and gills tended to be downregulated, and that in the gills after 24 h under heat stress was significantly lower than in the corresponding control group (Fig. 1B). Hemoglobin alpha gene expression also exhibited a significant decrease under the same condition (Supplementary Fig. 1A). We performed ISH to localize Hbß gene expression in the gill tissue after 24 h under heat stress (Fig. 2). Although the sense probe gave no blue staining signals (Fig. 2C and D), the gill tissues prepared from fish under heat stress gave weaker blue staining than that control group when the antisense probe was used (Fig. 2A and B): the signals from epithelial cells in the secondary gill filament were markedly decreased in the fish under heat stress.

### 3.2 Edwardsiella piscicida infection

**Fig. 3:**
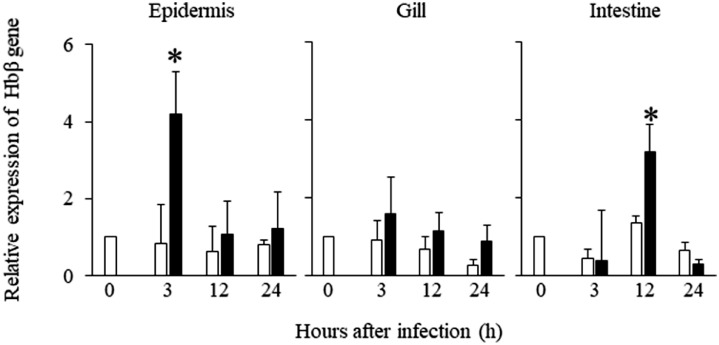
Changes of Hbβ gene expression in epidermis, gill, and intestine of Japanese flounder after *Edwardsiella piscicida* infection (closed bars) or immersed heart infusion broth (open bars). The gene expression levels were normalized to that of the housekeeping gene β-actin. Expression levels relative to the pre-infection control group (Pre) are shown. Values are means ± SD (*n* = 5). * *p* < 0.05 versus corresponding control group at the same time point (Mann-Whitney *U* test).

**Fig. 4:**
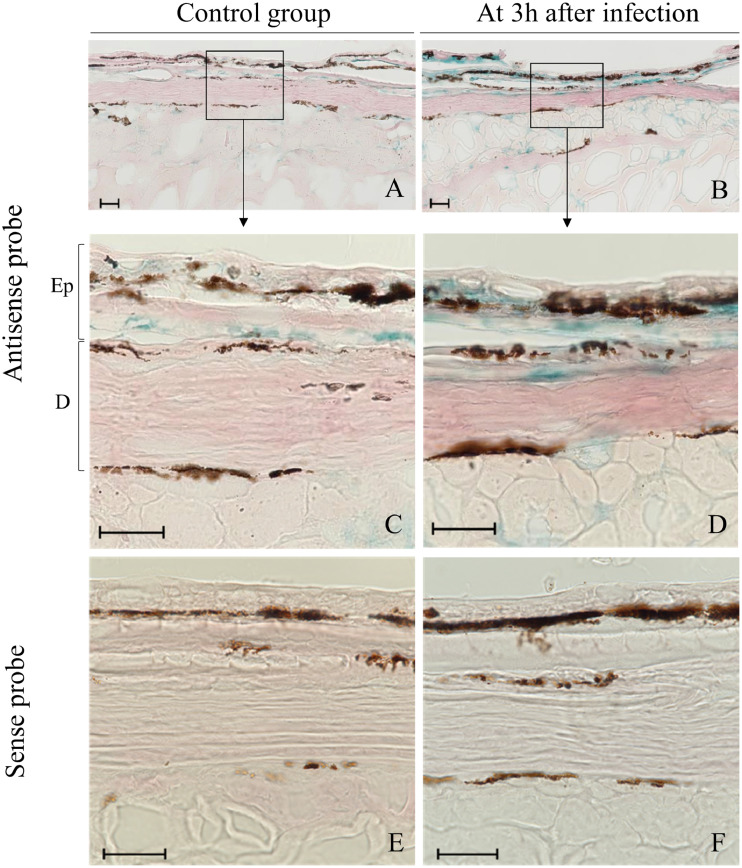
Hbβ mRNA expression (blue staining) visualized by *in situ* hybridization (ISH) (A–F) in skin tissue (ocular side) of Japanese flounder after infection with *Edwardsiella piscicida*. The sections were prepared from skin in the control group (A, C, and E) and at 3 h after infection (B, D, and F). ISH was performed by using the antisense probe (A–D) and the sense probe (E and F). C and D are higher magnification images of A and B, respectively. Scale bars, 10um. Epidermis (Ep), Dermis (D).

To investigate whether Hbß gene expression levels in the mucosal tissues of Japanese flounder were affected by *E. piscicida* infection, we serially collected mucosal tissues from infected fish and carried out qRT-PCR analysis. Hbß gene expression in the epidermis was significantly increased (by 4.2 times)-at 3 h after infection compared with the control group (pre-infection) (Fig. 3). Otherwise, there were no significant differences between the infection and control groups. Hemoglobin alpha gene expression did not exhibit a significant difference under the same condition (Supplementary Fig. 1B). In the gill tissue, gene expression tended to be higher in the infected group than in the control group during the experimental period (Fig. 3), although these differences were not statistically significant. In the intestine, gene expression was significantly increased at 12 h after infection compared with the control group (Fig. 3). In response to the results of the qRT-PCR analysis, we compared the localization of epidermis Hbß gene expression at 3 h after infection between the infection and control groups by using ISH (Fig. 4). The abundance and intensity of signaling cells in the epidermal and dermal layers were greater in the infection group than in the control group (Fig. 4C and D). The sense probe gave no staining signals in any skin section in either group (Fig. 4E and F).

### 3.3 Immunostimulant administration

**Fig. 5:**
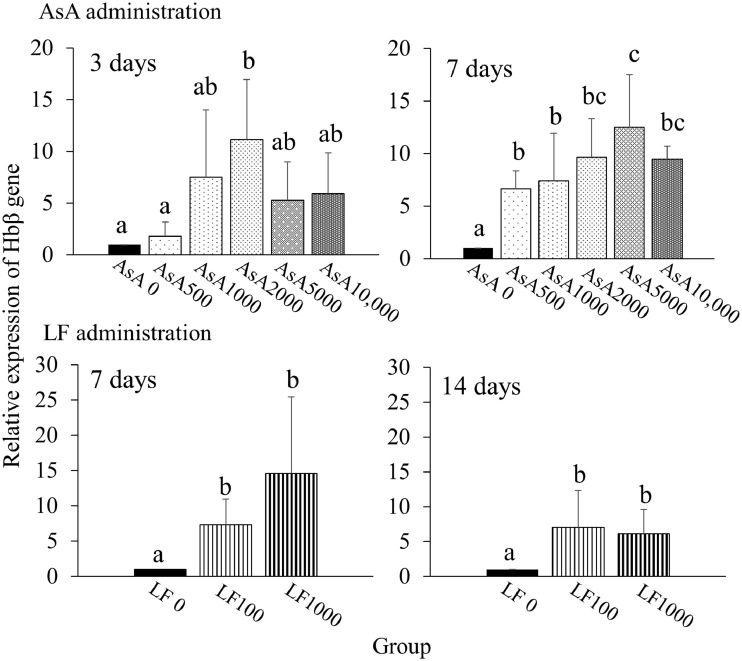
Effects of administration of various concentrations of ascorbic acid (AsA) or lactoferrin (LF) on Hbβ gene expression in the epidermis (ocular side) of Japanese flounder. AsA was administered by feeding trials for 3 and 7 days, and LF was administered by feeding trials for and 7 and 14 days. Gene expression levels were normalized to that of the housekeeping gene β-actin and are presented relative to the AsA0 or LF0 group (means ± SD, *n* = 5). Different letters on the bars indicate significant differences (*p* < 0.05, Tukey-Kramer multiple comparison test).

**Fig. 6:**
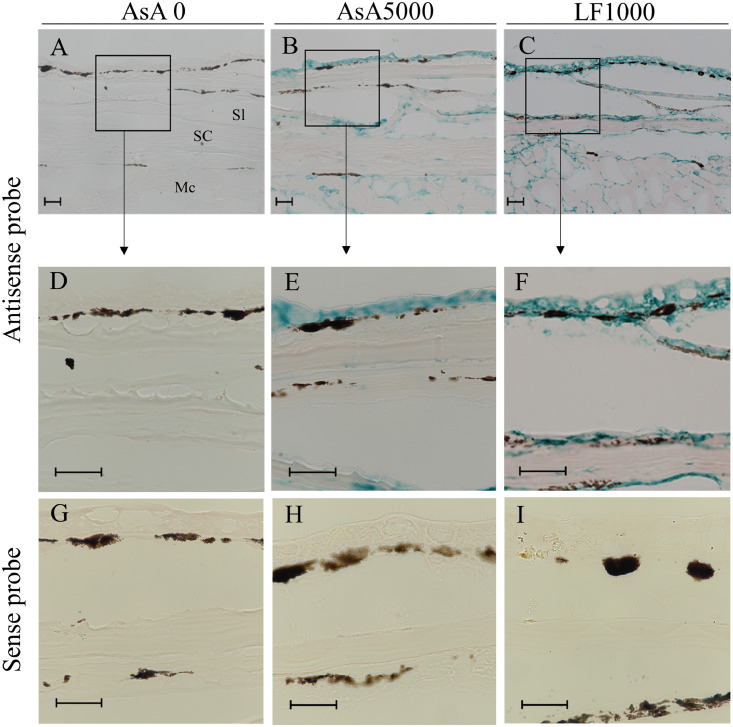
Hbβ mRNA expression (blue staining) visualized by *in situ* hybridization (ISH) (A–F) in skin tissue (ocular side) of Japanese flounder in AsA0, AsA5000 and LF1000 groups The sections were prepared from skin of the fishes fed commercial diet (A, D, and G) or experimental diet with either ascorbic acid (5000 mg/kg diet: B, E, and H) or lactoferrin (1000 mg/kg diet: C, F, and I) for 7 days. ISH was performed using the antisense probe (A–F) or sense probe (G–I). D–F are higher magnifications of A–C, respectively. Scale bars, 10 μm. Stratum laxum (Sl), stratum compactum (SC) and muscle cell (Mc).

To evaluate whether Hbß gene expression level could be modulated by different concentrations of AsA or LF, we performed qRT-PCR analysis. Although on day 7 of the feeding trials Hbß gene expression in the ocular-side epidermis was significantly greater (by 6.6–12.5 times) in all AsA groups than in AsA0, on day 3 the only significant increase compared with AsA0 was that of the AsA 2000 group (Fig. 5). In contrast, Hbß gene expression levels in both LF-fed groups were significantly greater (by 6.1–14.6 times) than in the LF0 group throughout the feeding trials (Fig. 5). Hemoglobin alpha gene expression did not exhibit a significant difference under the same condition (Supplementary Fig. 2). Concerning these qRT-PCR results, we next performed ISH to visualize the localization of Hbß gene expression in the skin tissues of the control (AsA0), AsA5000, and LF1000 groups (Fig. 6). Hbß gene expression in the superficial and basal cell layers of the epidermis was much higher in the AsA5000 and LF1000 groups than in the AsA0 group (Fig. 6D–F). In addition, clearly signaling cells in the stratum laxum, stratum compactum, and muscle were observed in both the AsA5000 group and the LF1000 group (Fig. 6A–C). In contrast, the sense probe gave no blue staining signals (Fig. 6G–I).

## 4 Discussion

In fish, a variety of physiological and immunological molecules, such as immunoglobulins, complement, lectins, ubiquitin, fatty-acid binding proteins, and antimicrobial peptides are secreted from the mucosal tissues: skin, gills, and intestine [19], [20], [21]. As the antimicrobial activity and secretion of these molecules can be affected by exposure to stress conditions such as high rearing density and high-water temperature, alterations of both factors are thought to be closely related to fish health condition and disease occurrence [22]. In the present study, we focused on Hbβ gene and analyzed its temporal expression upon heat treatment, bacterial infection, and immunostimulant administration.

Heat stress, a common stressor at fish farms and aquariums, can suppress immunity and other physiological functions in fish [23]. For example, elevation in water temperature can cause epithelial damage which an provoke respiratory disorders in the gill, a portal of entry for bacteria [[24],[25]]. Moreover, increasing in water temperature can lead to a decrease in oxygen solubility because the dissolved oxygen concentration is dependent on the water temperature [26]. We raised the water temperature from 20 to 30°C for heat stress and examined the gene expression levels of Hbβ and of HSP70, which is used to evaluate heat stress in fish. As HSP70 gene expression levels under heat stress increased by 10.1–35,244.4 times during the experimental period, we considered that the fish had been affected by heat stress. In contrast, Hbβ gene expression in the epidermis and gills decreased by 0.04–0.6 times after 24 h under heat stress condition; decrement of the signaling cells was observed in the epithelial cells of the gill secondary lamellae as well. These results suggest that heat stress affects the gill tissues especially the secondary lamellae; the decrease in Hbβ may cause respiratory disorders and infection of the gill tissue.

In fish, the antimicrobial activity of complement factor C3, lysozyme, and cathepsins B and L in the mucus increases after bacterial infection [[27],[28]]. Similarly, we found that Hbß gene expression in the epidermis and intestine also increased and then decreased after infection. Tort [22] reported that fish exposed to a stressor undergo an activating phase in which the innate immune response was enhanced. As Hbβ signaling cells were clearly observed by using ISH in the epidermis after infection, we suggest that Hbβ secretion increases upon infection and the derived oligopeptides with antimicrobial activity act against bacteria invading the fish's mucosal tissues.

Aquafarms and aquariums use a variety of immunostimulants that enhance stress resistance and innate immunity [[29],[30]]. However, there is a lack of knowledge of the correlation between immunostimulants and mucosal tissues in fish. Therefore, we treated the fish with high-concentration AsA and LF, which had been confirmed to improve stress resistance and survival in the face of viral and bacterial infections [[16], [17], [18], [30]]. For example, it has been reported that Asian catfish showed an increment in resistance to *Aeromonas hydrophila* upon feeding a diet supplemented with 100 mg LF/kg diet for 7days[17]. In the case of amberjack, Yokoyama et al. observed the improvement in resistance to low-salinity stress and *Neobenedenia girellae* infection upon feeding a diet supplemented with 1000 mg LF/kg diet for 14days [18]. Ishikawa et al. [15] showed that feeding rainbow trout with 5000 mg AsA/kg diet for 7days has led to fewer infection of hematopoietic necrosis virus. Lin and Shiau [31] showed that giving 400 mg AsA/kg diet to juvenile grouper for 7days resulted in an increment in resistance to *Vibrio carchariae* infection. In a previous study, we revealed that Hbß levels increased in the epidermis of Japanese flounder given AsA (2000 mg AsA/kg diet) [8]. Here we evaluated how epidermal Hbß gene expression could be modulated by different concentrations of AsA or another immunostimulant, LF. On day 7 of the feeding trials, high levels of expression of the Hbβ gene in epidermal were found in fish given AsA at 2000 mg (or more)/kg diet and in both LF-fed groups. Furthermore, ISH revealed numerous cells with strong Hbβ mRNA signals in the epidermis and dermis layer of fish given AsA or LF. As AsA administration endows fish with high tolerance to heat stress [[16],[31]], the increase in Hbβ—an oxygen-carrying protein—in the epidermis and dermis layer may be related to the enhancement of high-water temperature tolerance. In addition, as AsA or LF administration markedly enhances resistance against pathogens in fish [12], we suggest that increased epidermal levels of antimicrobial peptides derived from Hbβ contribute to this resistance.

Because fish health status is influenced by dietary nutrients [32] or stressors, including water temperature and pathogens [[33],[34]], numerous studies that can be referred to in fish farming and rearing research [35] have been conducted to search indicators for monitoring fish health condition. So far, plasma cortisol level has been used as an indicator of acute stress [36] and immune factors such as serum IgM level [37] and phagocytic activity of neutrophils and macrophages [[38],[39]] have been used as health indicators in fish. Our results showed that expression of the Hbß gene was decreased in the epidermis and gills by heat stress and increased in the epidermis by immunostimulant administration. These results suggest that Hbβ in mucosal tissues could be used as an indicator for checking fish health status. Clearly, we have to compare Hbβ with other health indicators to confirm its efficacy in fish.

In conclusion, our present study revealed alteration of Hbß gene expression in mucosal tissues of Japanese flounder changed in response to heat stress, bacterial infection and immunostimulant administration. The results indicate that Hbß gene but not Hb alpha gene is involved in a defensive mechanism against heat stress and bacterial infection found in fish mucosal tissues. Using mucosal tissues, which can be easily collected as samples, Hbß gene expression could be introduced as an indicator to monitor both fish health condition and effectiveness of immunostimulants.

## Declaration of Competing Interest

The authors declare that they have no known competing financial interests or personal relationships that could have appeared to influence the work reported in this paper.

## Appendix Supplementary materials

Supplementary Fig. 2 Effects of administration of various concentration of ascorbic acid (AsA) or lactoferrin (LF) for 7days on Hb alpha and Hbβ gene expression in the ocular side of epidermis of Japanese flounder. The gene expressions were normalized to that of the housekeeping gene β-actin and are presented relative to the AsA0 or LF0 group (means ± SD, *n* = 5). The difference of Hb alpha and Hbβ gene expression levels in various concentration of AsA or LF were analyzed by Steel-Dwass and Tukey-Kramer test, respectively. Image, image 1

Image, image 2

Image, application 3
