# Supplementary material for: Alteration of hemoglobin ß gene expression in mucosal tissues of Japanese flounder, Paralichthys olivaceus, in response to heat stress, Edwardsiella piscicida infection, and immunostimulants administration
Source: Fish Shellfish Immunol Rep. 2022 Jan 8;3:100049. doi: 10.1016/j.fsirep.2021.100049 (PMC9680101; doi:10.1016/j.fsirep.2021.100049)
Supplement: Supplementary file 1 — Supplementary Fig. 2 Effects of administration of various concentration of ascorbic acid (AsA) or lactoferrin (LF) for 7days on Hb alpha and Hbβ gene expression in the ocular side of epidermis of Japanese flounder. The gene expressions were normalized to that of the housekeeping gene β-actin and are presented relative to the AsA0 or LF0 group (means ± SD, n = 5). The difference of Hb alpha and Hbβ gene expression levels in various concentration of AsA or LF were analyzed by Steel-Dwass and Tukey-Kramer test, respectively. [file mmc1.pptx]

## Slide 1
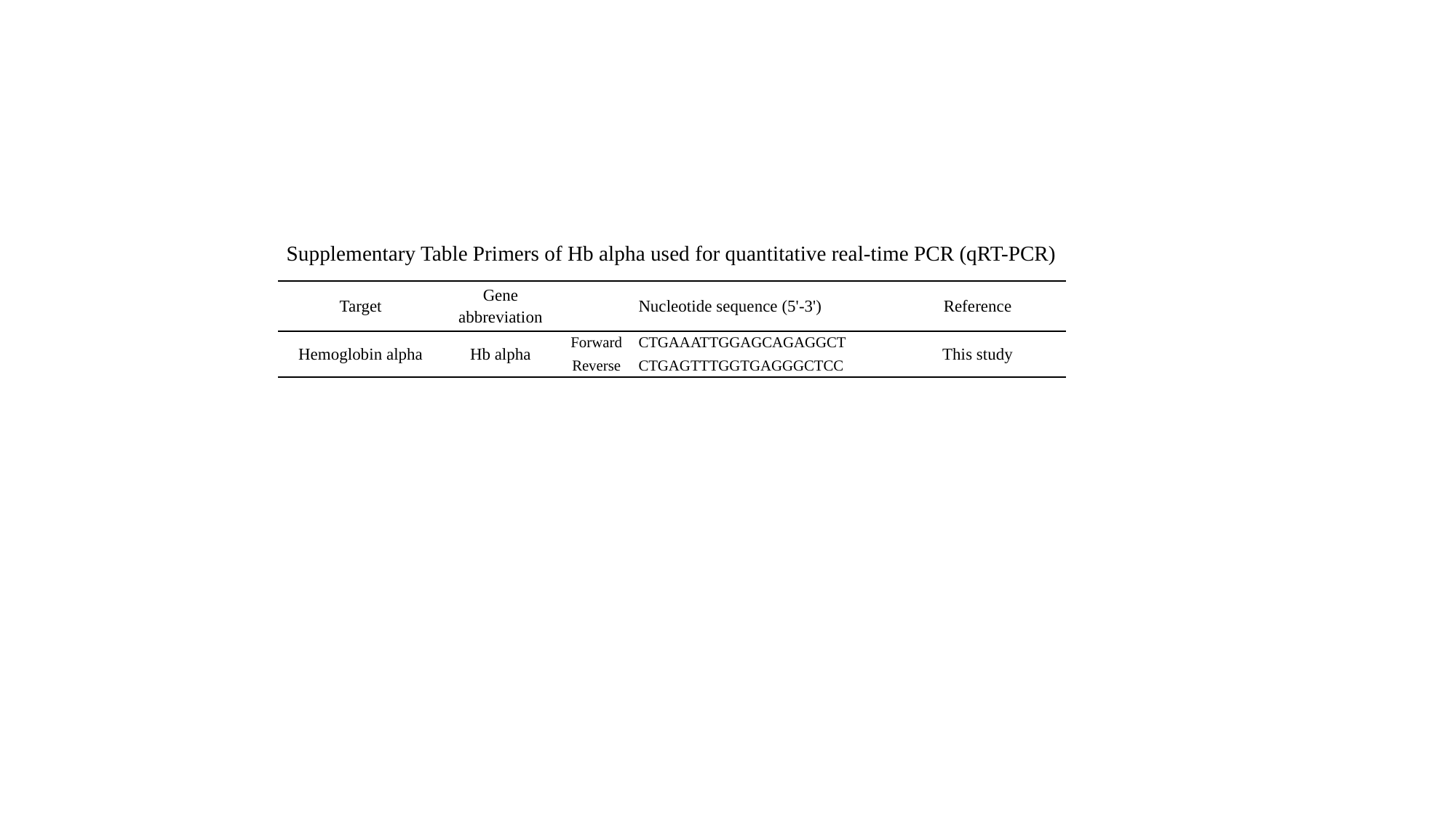

Supplementary Table Primers of Hb alpha used for quantitative real-time PCR (qRT-PCR)
| Target | Gene abbreviation | | Nucleotide sequence (5'-3') | Reference |
| --- | --- | --- | --- | --- |
| Hemoglobin alpha | Hb alpha | Forward | CTGAAATTGGAGCAGAGGCT | This study |
| | | Reverse | CTGAGTTTGGTGAGGGCTCC | |
