# Supplementary material for: Alteration of hemoglobin ß gene expression in mucosal tissues of Japanese flounder, Paralichthys olivaceus, in response to heat stress, Edwardsiella piscicida infection, and immunostimulants administration
Source: Fish Shellfish Immunol Rep. 2022 Jan 8;3:100049. doi: 10.1016/j.fsirep.2021.100049 (PMC9680101; doi:10.1016/j.fsirep.2021.100049)

Supplementary figure 1 Hemoglobin alpha and Hbβ gene expression in the gill tissues under heat stress (A) and in the ocular side of epidermis after infection (B) of Japanese flounder were determined by quantitative real-time PCR (n=5). The gene expression levels were normalized to that of the housekeeping gene β-actin. Values are means ± S.D. * p < 0.05 (Mann-Whitney *U* test).


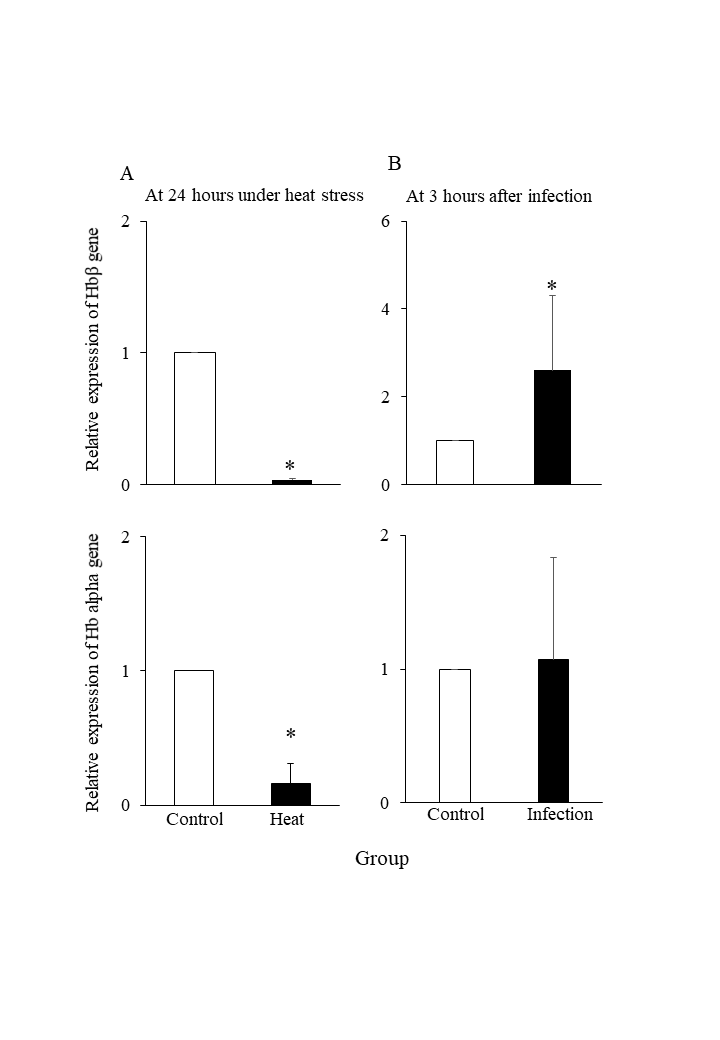

Supplement: Supplementary file 2 [file mmc2.docx]
